# 高分辨熔解曲线法检测非小细胞肺癌*p53*基因的突变

**DOI:** 10.3779/j.issn.1009-3419.2011.10.01

**Published:** 2011-10-20

**Authors:** 志红 陈, 社娟 安, 至 谢, 红虹 严, 剑光 陈, 健 苏, 绪超 张, 飞玉 牛, 伟浜 郭, 一龙 吴

**Affiliations:** 510080 广州，广东省人民医院医学研究中心，广东省医学科学院，广东省肺癌研究所 Medical Research Center, Guangdong General Hospital, Guangdong Lung Cancer Institute, Guangdong Academy of Medical Sciences, Guangzhou 510080, China

**Keywords:** *p53*基因, 突变, 测序, *p53* gene, Mutation, Sequence

## Abstract

**背景与目的:**

*p53*基因与人类多种肿瘤相关，突变型具有致癌作用，主要分布在外显子5-8。本研究旨在建立高分辨熔解曲线（high resolution melting, HRM）检测非小细胞肺癌（non-small cell lung cancer, NSCLC）患者*p53*基因突变的方法，探讨*p53*基因突变的特点及其在NSCLC发生发展中的演变规律。

**方法:**

采用HRM法检测264例NSCLC患者肿瘤组织和54例癌旁肺组织*p53*基因外显子5-8的突变，突变样品进一步使用PCR产物直接测序法分析确定突变类型；HRM法检测阳性而PCR产物直接测序法检测阴性的样品，进一步进行亚克隆测序证实。

**结果:**

54例对照未发现突变。264例肿瘤组织中，HRM法检出*p53*基因突变104例，102例得到PCR产物直接测序法证实，突变率为39.4%；95例为点突变，7例为碱基插入和缺失导致的移码突变。p53外显子5-8的突变率分别为11.7%、8%、12.5%和10.6%，差异无统计学意义（*P*=0.35）。*p53*基因突变与性别有关，与其它临床病理特征无关。

**结论:**

HRM法筛选*p53*基因突变样品，具有操作简便、快速、敏感、单管避免污染等优点，值得推广。*p53*基因的突变特点提示，*p53*基因突变是自发性突变，可能是DNA合成和修复过程中的随机错误所致。

## Abstract (en)

**Background and objective:**

It has been proven that *p53* gene was related to many human cancers. The mutations in *p53* gene play an important role in carcinogensis and mostly happened in exon 5-8. The aim of this study is to establish a high resolution melting (HRM) assay to detect *p53* mutations from patients with non-small cell lung cancer (NSCLC), to investigate the characteristics of *p53* gene mutations, and to analyze the relationship between *p53* mutations and evolution regularity of pathogenesis.

**Methods:**

*p53* mutations in exon 5-8 were detected by HRM assay on DNA insolated from 264 NSCLC samples derived from tumor tissues and 54 control samples from pericancerous pulmonary tissues. The mutation samples by the HRM assay were confirmed by sequencing technique. Samples which were positive by HRM but wild type by sequencing were further confirmed by sub-clone and sequencing.

**Results:**

No mutation was found in 54 pericancerous pulmonary samples by HRM assay. 104 of the 264 tumor tissues demonstrated mutation curves by HRM assay, 102 samples were confirmed by sequencing, including 95 point mutations and 7 frame shift mutations by insertion or deletion. The mutation rate of *p53* gene was 39.4%. The mutation rate from exon 5-8 were 11.7%, 8%, 12.5% and 10.6%, respectively and there was no statistically significant difference between them (*P*=0.35). *p53* mutations were significantly more frequent in males than that in females, but not related to the other clinicopathologic characteristics.

**Conclusion:**

The results indicate that HRM is a sensitive in-tube methodology to detect for mutations in clinical samples. The results suggest that the arising p53 mutations in NSCLC may be due to spontaneous error in DNA synthesis and repair.

*p53*基因是迄今为止发现的与人类多种肿瘤相关性最高的基因。野生型*p53*基因是一个肿瘤抑制基因，而其突变型则具有致癌作用，*p53*基因突变广泛分布外显子5-8，可达95%-98%^[1, 2]^。但p53的突变热点较多，比较分散，目前通常采用组织DNA直接测序法检测*p53*基因突变，费时且昂贵，无法满足临床需要。本研究旨在建立高分辨熔解曲线（high resolution melting, HRM）检测非小细胞肺癌（non-small cell lung cancer, NSCLC）患者*p53*基因突变的方法^[3, 4]^，探讨*p53*基因突变的特点及其在NSCLC发生发展中的演变规律。

## 1 材料与方法

### 1.1 标本来源

病例组为2007年-2009年广东省人民医院的NSCLC患者肿瘤标本，共264例，均为汉族，男184例，女80例，年龄23岁-88岁，均经病理学诊断确诊，且在知情同意后签署了知情同意书。对照组为肺癌患者的癌旁组织标本，共54例，均为汉族，男38例，女16例，年龄28岁-62岁。

### 1.2 主要试剂与仪器

HRM分析必需试剂：LightCycler 480 High Resolution Melting Master（瑞士Roche公司）；其它试剂和仪器：DNA抽提试剂盒（上海华舜公司）、pGEM-T（美国Promega公司）、Gel Extraction Kit（德国Qiagen公司）、BIGDYE（美国Applied Biosystems公司）、核酸蛋白测定仪（德国Eppendorf公司）、PCR扩增仪（美国BD公司）、LightCycler 480荧光定量分析仪（瑞士Roche公司）、ABI3100测序仪（美国Applied Biosystems公司）。

### 1.3 方法

#### 1.3.1 标本采集和DNA提取

组织在手术切除后快速冻存于液氮中，-80 ℃保存待用。将冰冻切片评估肿瘤组织含量>50%的标本用于检测。组织样本（50 mg）中的DNA提取按照基因组DNA抽提试剂盒说明书操作。使用Eppendorf核酸蛋白测定仪测定DNA纯度及含量，要求吸光度（A）值280/260>1.80，调整DNA浓度至5 ng/μL。

#### 1.3.2 HRM检测

按照文献^[5]^设计*p53*基因突变检测引物，外显子5-8的引物序列见表 1。所有引物均由大连宝生物公司合成，用HPLC进行纯化。PCR体系包括10 ng的基因组DNA、1xPCR Master mix、3 mmol/L MgCl_2_、250 nmol/L的正反向引物，并用PCR级别的水补足至20 μL。所有的PCR均重复2次。PCR和HRM分析均在LightCycler 480荧光定量分析仪上进行。PCR条件：95 ℃ 10 m，95 ℃ 10 s，60 ℃ 15 s，72 ℃ 25 s，45个循环。HRM分析条件：95 ℃ 1 min，40 ℃ 1 min，熔解曲线数据收集从65 ℃到95 ℃温度上升率为1 ℃/s，且每升高1 ℃进行25次数据采集。

**1:** p53 HRM引物表 p53 HRM primers

Exon		Sequence (5’ to 3’)	Amplicon size
Exon 5	Forward	tgttcacttgtgccctgact	268 bp
	Reverse	cagccctgtcgtctctccag	
Exon 6	Forward	gcctctgattcctcactgat	181 bp
	Reverse	ttaacccctcctcccagaga	
Exon 7	Forward	actggcctcatcttgggcct	171 bp
	Reverse	tgtgcagggtggcaagtggc	
Exon 8	Forward	taaatgggacaggtaggacc	230 bp
	Reverse	tccaccgcttcttgtcctgc	
HRM: high resolution melting.			

#### 1.3.3 *p53*基因突变型和野生型（wild-type, wt）质粒的构建

采用TA克隆法分别构建*p53*基因外显子5-8的野生型克隆和突变型克隆，即分别以p53外显子5-8的测序结果为阳性的标本的DNA和测序结果为阴性的标本的DNA为模板进行PCR。PCR产物采用凝胶回收试剂盒进行回收纯化，纯化产物与pGEM-T载体连接，转化入大肠杆菌感受态细胞。37 ℃过夜培养，筛选出重组体。将重组体加至细菌Luria-Bertani（LB）培养基，37 ℃摇床孵育过夜，提取质粒、测序，验证转入序列的准确性。序列正确的质粒-20 ℃保存待用。

#### 1.3.4 HRM分析的灵敏性试验

野生型和突变型质粒的DNA浓度均调整至5 ng/μL，然后按不同比例将二者混合，使得样本中突变型质粒所占的比例分别为2%、5%、10%、20%、50%、100%，各取1 μL混合质粒DNA作为模板用于HRM检测。

#### 1.3.5 直接测序法检测

HRM分析后，阳性标本采用测序引物进行PCR扩增，PCR产物按试剂盒操作说明书切胶过柱纯化，以纯化后的PCR产物为模板，在ABI3100测序仪上按测序试剂盒按照说明书进行测序检测。采用Chromas软件分析测序图谱，判读*p53*基因外显子5-8的突变类型。

#### 1.3.6 亚克隆测序

HRM法检测阳性而PCR产物直接测序法检测阴性的样品，进一步进行亚克隆测序证实。

### 1.4 统计学分析

SPSS 13.0统计软件分析数据，采用卡方检验进行分析。*P* < 0.05为差异有统计学意义。

## 2 结果

### 2.1 HRM法的检测灵敏性

如图 1所示，HRM法检测p53不同外显子不同比例的系列混合质粒DNA，2次结果的重复性好，且可检测出仅含2%-10%突变型DNA混合样本中的突变，提示HRM法检测p53基因突变的灵敏度可达2%-10%。

**1:**
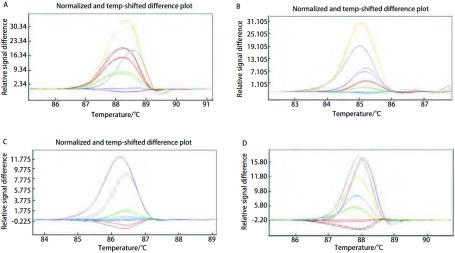
HRM法检测*p53*基因外显子5-8的敏感性结果。野生型和突变型质粒混合后突变型所占的比例分别为2%、5%、10%、20%、50%和100%，分别显示不同颜色的熔解曲线。A：外显子5：蓝（wt），绿（2%），红（5%），棕（10%），黄（20%），灰（50%），紫（100%）；B：外显子6：蓝（wt和2%），绿（5%），红（10%），紫（20%），黄（50%），灰（100%）；C：外显子7：蓝（wt，2%和5%），绿（10%），灰（20%），紫（50%），红（100%）；D：外显子8：红（wt），绿（2%），蓝（5%），黄（10%），紫（20%），灰（50%），棕（100%） The exon 5-8 of *p53* gene sensitive analysis by HRM. The mutation plasmid DNA was mixed with wild-type plasmid DNA to dilute the mutant allele to 2%, 5%, 10%, 20%, 50% and 100% of the total alleles. The melting curves of each dilution are shown in different color. A: exon 5: blue (wt), green (2%), red (5%), brown (10%), yellow (20%), grey (50%) and purple (100%); B: exon 6: blue (wt and 2%), green (5%), red (10%), purple (20%), yellow (50%) and grey (100%); C: exon 7: blue (wt, 2% and 5%), green (10%), grey (20%), purple (50%) and red (100%); D: exon 8: red (wt), green (2%), blue (5%), yellow (10%), purple (20%), grey (50%) and brown (100%)

### 2.2 HRM法和测序法检测结果

HRM法检测54例对照组的正常组织，未能检出*p53*基因突变。264例NSCLC患者的组织，检出具有*p53*基因突变104例，102例经测序法得到证实，突变率为39.4%，1例经亚克隆测序为野生型，1例DAN量不够无法进行亚克隆分析；95例为点突变，其中错义突变74例，无义突变6例，同义突变15例，其余7例为碱基插入和缺失导致的移码突变，突变中碱基转换突变占总突变的93.1%。各外显子不同突变类型的HRM曲线及测序结果见图 2-图 5。

**2:**
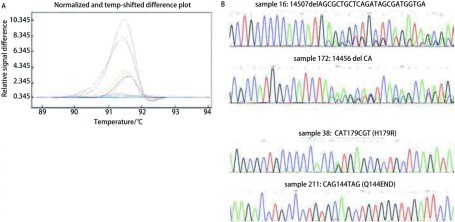
*p53*外显子5不同突变类型样品的HRM曲线和测序结果。A：HRM曲线图。蓝（野生型），灰（样品16），紫（样品172），黄（样品38），棕（样品211）；B：测序结果 HRM difference plot and sequence data for some mutation samples for p53 exon 5. A: Difference plot of some samples. Blue (wt), grey (sample 16), purple (sample 172), yellow (sample 38) and brown (sample 211); B: Sequencing traces for sample16, 172, 38 and 211

**3:**
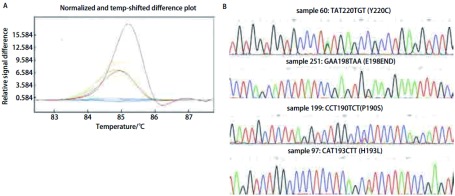
*p53*外显子6不同突变类型样品的HRM曲线和测序结果。A: HRM曲线图。蓝（野生型），棕（样品60），黄（样品251），绿（样品199），红（样品97）；B：测序结果 HRM difference plot and sequence data for some mutation samples for p53 exon 6. A: Difference plot of some samples. Blue (wt), brown (sample 60), yellow (sample 251), green (sample 199) and red (sample 97); B: Sequencing traces for sample 60, 251, 199 and 97

**4:**
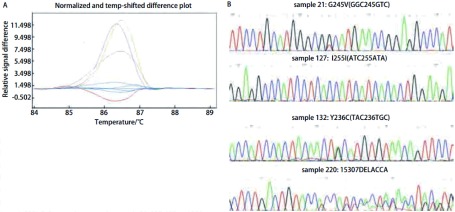
*p53*外显子7不同突变类型样品的HRM曲线和测序结果。A: HRM曲线图。蓝（野生型），绿（样品21），紫（样品127），灰（样品132），红（样品220）；B：测序结果 HRM difference plot and sequence data for some mutation samples for p53 exon 7. A: Difference plot of some samples. Blue (wt), green (sample 21), purple (sample 127), grey (sample 132) and red (sample220); B: Sequencing traces for sample 21, 127, 132 and 220

**5:**
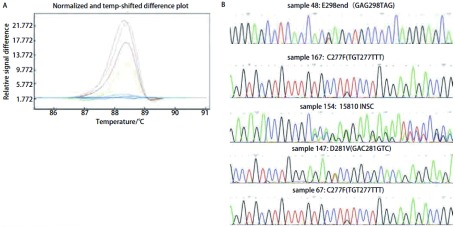
*p53*外显子8不同突变类型样品的HRM曲线和测序结果。A: HRM曲线图。蓝（野生型），紫（样品48），灰（样品167），棕（样品154），绿（样品147），黄（样品67）；B：测序结果 HRM difference plot and sequence data for some mutation samples for p53 exon 8. A: Difference plot of some samples. Blue (wt), purple (sample 48), grey (sample 167), brown (sample 154), green (sample 147) and yellow (sample 67); B: Sequencing traces for sample 48, 167, 154, 147 and 67

### 2.3 p53外显子5-8的突变结果

p53外显子5、6、7、8的突变率分别为11.7%、8%、12.5%、10.6%，差异无统计学意义（*P*=0.35），且各外显子的突变率与吸烟、分化等临床参数均无关（*P*>0.05）。

### 2.4 *p53*基因突变与临床病理特征的关系（表 2）

**2:** *p53*基因突变与临床病理特征的关系 Correlation between *p53* gene mutations and clinicopathologic features

Clinical characteristics	*p53* gene		*χ*^2^	*P*
	Wild-type (*n*=160)	Mutation (*n*=104)		
Gender			4.243	0.039
Male	104	80		
Female	56	24		
Age(yr)			0.181	0.670
≤60	95	59		
>60	65	45		
Smoking status			1.952	0.162
Smoker	94	52		
Non-smoker	66	52		
Histology			0.470	0.493
Adenocarcinoma	117	72		
Non-adeno	43	32		
TNM stage			0.023	0.879
Ⅰ+Ⅱ	13	9		
Ⅲ+Ⅳ	147	95		
Differentiation^*^			0.267	0.698
High	120	78		
Moderate	31	18		
Low	9	7		
^*^One sample hasn’t the differentiation data.				

*p53*基因突变与性别有关，男性突变率（43.5%）明显高于女性（30%）（*P*=0.039）。*p53*基因突变与年龄、吸烟、病理、分期和分化等临床病理特征均无关（*P*>0.05）。

## 3 讨论

抑癌基因*p53*的突变已被证实与人类半数以上的肿癌发生有关。该基因编码一种分子量为53 kDa的蛋白质，命名为p53。一旦*p53*基因发生突变，p53蛋白失活，细胞分裂失去节制发生癌变。目前对*p53*基因变异与肿癌生物学行为关系的研究发现，在肺癌、胃癌、乳腺癌及大肠癌中，具有p53突变患者的恶性程度较高、预后较差^[2, 6, 7]^。本研究采用HRM结合DNA序列分析法在39.4%的NSCLC癌组织中检出p53突变，且突变与临床分期和分化程度均无关系，提示*p53*基因突变可能于肺癌早期就发生，并持续于肿瘤发展的全过程，有助于判断NSCLC患者的预后。*p53*基因有多种突变类型，主要为点突变，导致碱基转换，发生错义突变，本研究结果与Lee等^[8]^的报道一致。Suzuki等^[9]^报道*p53*基因突变与吸烟有相关性，本研究结果显示*p53*基因突变虽然在吸烟者中较多见，但吸烟与非吸烟患者间无统计学意义。*p53*基因突变在男性中的发生率明显高于女性，这与Lee等^[8]^报道非吸烟女性中*p53*基因突变的发生率较低相一致。*p53*基因突变的突变特点和分布等均无统计学意义，提示*p53*基因突变是自发性突变，可能是在DNA合成和修复过程中的随机错误所致。

本研究共发现7例*p53*基因由于碱基插入和缺失导致的移码突变，HRM法和测序法均可确定。其测序图谱部分为单一峰，从突变位置开始为重叠峰。为确定是含有杂合突变还是由于PCR错配而导致的重叠峰，可采用高保真酶重复进行PCR和测序，如出现重叠峰的位置均一致，可判断为杂合突变，因为PCR错配的发生点不是唯一的，每次均发生在同一点的几率较少，或者进行克隆性测序。

目前检测基因突变的方法有很多，最常用的有直接测序法、荧光探针法、单链构象多态（single-strand conformation polymorphism, SSCP）法、变性高效液相色谱法（denaturing high performance liquid chromatography, DHPLC）等，各种方法均有不同的优缺点。直接测序法由于能直接鉴别出具体突变的碱基，所以一直都被作为检测突变的金标准。但这种方法步骤繁琐，耗时长，费用高，敏感性相对低，难以达到临床上的需要。本研究使用的HRM法是近几年来在国内外兴起的最新的单核苷酸多态性（single-nucleotide polymorphism, SNP）及突变研究的工具。它是通过熔解温度（Tm）的迁移而产生不同形状的熔解曲线来区分不同的基因型。这种方法不受突变碱基位点与类型的局限，无需根据不同的序列设计特异性探针，在PCR结束后直接运行高分辨率熔解，就可完成对样品基因型的分析。HRM法所使用的PCR扩增酶必须是热启动酶，减少非特异性扩增，使用的染料必须是饱和染料，才能根据熔解曲线的不同来区分不同的基因型。应用HRM法筛选NSCLC肿瘤标本的p53基因突变样品，具有操作简便、快速、敏感、单管避免污染等优点，完全符合临床个体化治疗的要求，值得推广。
